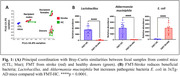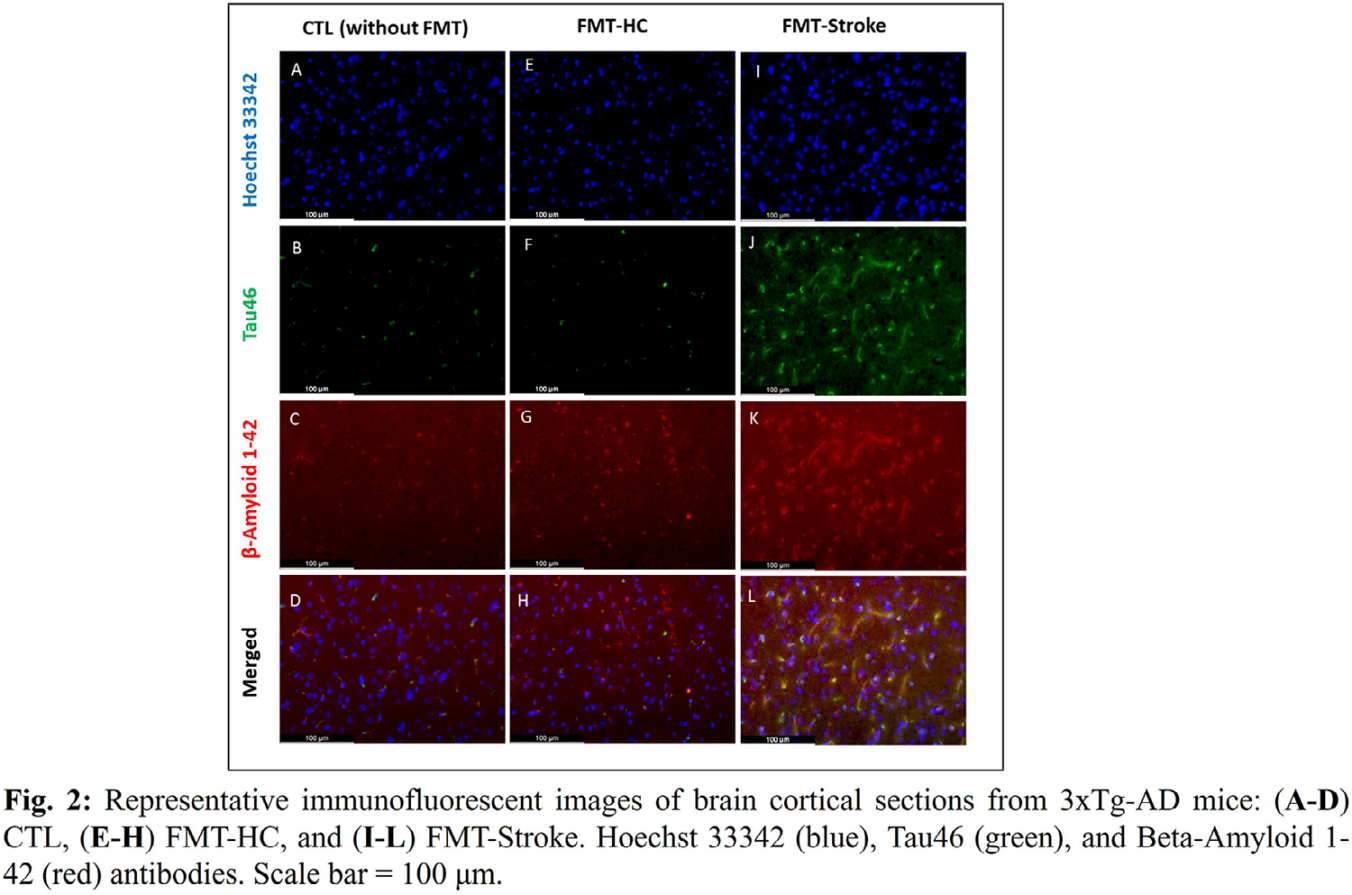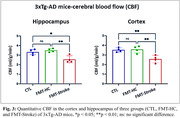# Fecal microbiome transplantation from stroke donors promotes Alzheimer’s disease brain pathology and hypoperfusion in the 3xTg‐AD mice

**DOI:** 10.1002/alz.092891

**Published:** 2025-01-03

**Authors:** Chetan Aware, Abeoseh Flemister, Ya‐Hsuan Chang, Aaron Ericsson, Zezong Gu, Jiankun Cui, Amitai Zuckerman, Lixin Ma, Ai‐Ling Lin

**Affiliations:** ^1^ University of Missouri, Columbia, MO USA; ^2^ University of Kentucky, Lexington, KY USA

## Abstract

**Background:**

The link between stroke and Alzheimer’s disease (AD) is recognized. However, the underlying mechanisms are not yet clear. This study seeks to determine if increased AD risk is linked to gut dysbiosis caused by acute ischemic stroke. Specifically, we examined whether fecal microbiome transplantation (FMT) from stroke patients to an AD mouse model would increase dysbiosis, reduce cerebral blood flow (CBF), and lead to increased levels of Aβ and tau of the mice.

**Method:**

Stool samples were collected from acute stroke patients (n = 8) and age‐matched healthy controls (HC) (n = 8) (age 55‐80 yrs.). Samples were transplanted orally to male (FMT‐Stroke, n = 14 and FMT‐HC, n = 12) and female (FMT‐Stroke, n = 17 and FMT‐HC, n = 15) 3xTg‐AD mice (3 months of age) for 4 weeks after receiving antibiotics treatment. Another group of naive mice (CTL) without FMT (M:F = 5:8) were included. Both fecal DNA of the donors and mice were sequenced by 16S rRNA. A subset of mice (n = 4/group) was sent for CBF measurements by 7T MRI, and Aβ and tau determination using immunohistochemistry (IHC).

**Result:**

Bray‐Curtis analysis on beta diversity revealed that stroke and HC donors, as well as recipient mice and CTL mice differ significantly from each other with strong sex differences (Fig. 1A). FMT‐Stroke‐treated mice showed decreased SCFA‐producing bacteria (Lactobacillus and Akkermansia muciniphila) and increased pathogenic bacteria (E. coli) compared to FMT‐HC mice (Fig. 1B). Further, the FMT‐Stroke group exhibited elevated levels of tau and Aβ in the cortex compared to the CTL and FMT‐HC groups (Fig. 2). MRI study demonstrated that FMT‐Stroke manifested in lower CBF in the cortex and hippocampus compared with the other two groups (Fig. 3).

**Conclusion:**

Our study provides evidence that gut dysbiosis following a stroke may actively contribute to the development of AD pathology and decrease CBF, thereby increasing the risk of AD. Addressing gut dysbiosis through dietary changes or medication could be crucial in helping stroke survivors reduce their risk of developing AD. These findings could pave the way for novel treatments aimed at lowering the risk of stroke‐associated AD.